# Late-life depression and the family physician

**DOI:** 10.4102/safp.v64i1.5534

**Published:** 2022-06-28

**Authors:** Sanushka Moodley, Alexandra Maisto

**Affiliations:** 1 Department of Psychiatry, Faculty of Health Sciences, School of Clinical Medicine, University of the Witwatersrand, Johannesburg, South Africa

**Keywords:** Late life, depression, older, geriatric mental health, major depressive disorder, antidepressants, cognitive impairment, dementia

## Abstract

Late-life depression (LLD) is a common disorder seen in clinical practice. Depression in this population group is often left undetected and untreated. The majority of elderly individuals who seek help present to the primary health care setting. The family physician is ideally placed to screen for symptoms of LLD, given that they often have longitudinal knowledge of the patient’s history, premorbid personality, functioning and overall health status. An understanding of risk factors, differential diagnoses, appropriate opportunistic screening tools and decision-making around management plans can assist the family physician in the early detection and treatment of these patients. In doing so, this may lead to a decrease in mortality and morbidity and enhance the patient’s quality of life.

## Introduction

Late-life depression (LLD) is defined as depression that occurs after the age of 60 years. The prevalence rate of LLD in the primary care setting is 6% – 9% and higher than that of community prevalence rates of 1% – 3%.^1^ This reaffirms that estimated prevalence rates vary depending on context in which a patient is being seen.^1^ The prevalence rises steeply when considering elderly individuals in old age homes and those with comorbid medical conditions or significant psychosocial stressors.^2^ Depression is a significantly debilitating illness. In the World Health Organization’s (WHO) Global Burden of Disease Study in 2019, mental disorders were found to be one of the leading causes of health-related disability worldwide, with depressive and anxiety disorders accounting for the majority of the burden.^2^ Indeed, the prevalence of depressive disorders has been further worsened by the coronavirus disease 2019 (COVID-19) pandemic.^3^

The majority of elderly individuals seek help in a primary care setting, and depression in this population group is often left undetected and untreated. Depression in elderly people significantly increases the risk of cognitive disorders, cardiovascular disorders such as cerebrovascular events and ischaemic heart disease, cancers and suicide. Depression is also widely established to worsen the outcome of medical comorbid conditions and interfere with treatment adherence in affected individuals. Improving health care providers’ knowledge and diagnostic capacity, addressing stigma and providing access to evidence-based treatment are urgently needed to improve medical outcomes and quality of life measures in this vulnerable population.

### Presentation and diagnostic challenges

Late-life depression may represent a continuation or relapse of a pre-existing depressive illness or may represent a new onset of mood symptoms. The core features of major depressive disorder are the same irrespective of the age of presentation; however, barriers to diagnosis in this age group may include more atypical presentations, especially in male patients where the prevailing mood may appear more angry and apathetic, with anhedonia rather than overt sadness. Individuals may also describe more somatic complaints rather than focus on their emotional experiences. They may minimise psychological problems as a result of underlying stigmatising opinions towards mental health. Caregivers, healthcare workers and patients themselves may have a misconception that these symptoms represent the normal process of ageing, and thus patients may not seek care.

Diagnosis may be further complicated by the presence of medical comorbidity, with or without a superimposed delirium or hypokinetic delirium, as well as polypharmacy. Importantly, polypharmacy may take the form of prescribed medication, over-the-counter medication or complementary and alternative medication (CAMS) such as medicinal marijuana. The cause of neurovegetative symptoms such as those of fatigue, anorexia, insomnia, psychomotor slowing and pain may be difficult to determine. It is important to differentiate if these symptoms represent physical illness, medication side effects or symptoms of depression. Comorbid cognitive deficits may also impact the capacity of individuals to accurately report or even identify their symptoms. Subthreshold symptoms are also common in this population group, and in addition to causing a significant functional impairment in itself, they may act as a significant risk factor for the development of major depressive disorder and suicidality. These challenges may reduce the chances of early diagnosis and delay access to appropriate care.

## An approach to assessment

One needs to have a high index of suspicion to consider LLD as a possible differential diagnosis or comorbidity in elderly patients.^12^ An assessment requires a good understanding of the underlying risk factors, familiarity with appropriate screening tools and a structured, thorough history to evaluate all relevant contributing factors and exclude appropriate differentials.

### Understanding risk factors

**FIGURE 1:**
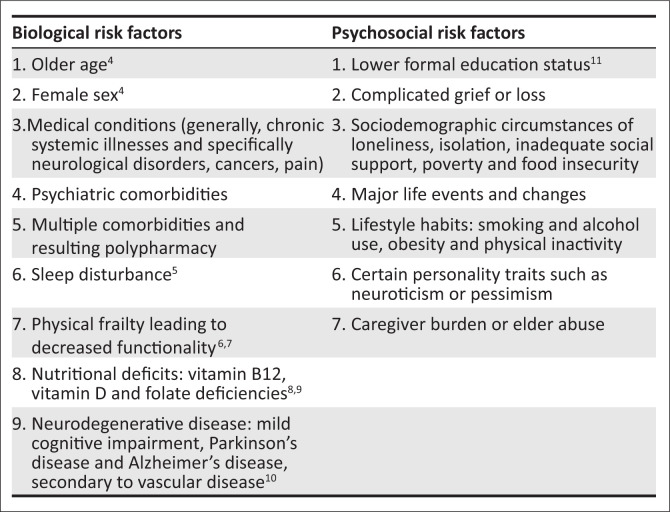
Risk factors for the development of late-life depression.

Understanding the risk factors of LLD allows for greater awareness, a higher index of clinical suspicion and increased sensitivity regarding making the diagnosis. As with many psychiatric disorders, risk factors can often be thought of in terms of biological and psychosocial risk factors, as seen in Figure 1. A knowledge of modifiable risk factors may help in the prevention of LLD in patients.

### Screening

Opportunistic screening for depressive symptoms in the at-risk patient can assist in early diagnosis, and subsequent appropriate management improves the quality of life and decreases morbidity and mortality.

Reliable opportunistic screening tools include:

- Patient Health Questionnaire (PHQ2)
  ■ This tool takes less than 1 min to administer and has a sensitivity of 100% and specificity of 70%.^13^
- Geriatric Depression Scale (GDS)
  ■ This tool takes less than 5 min to administer and is validated in > 80-year-olds with severe cognitive impairment (Mini-Mental Status Examination score > 10). It is also the preferred screening tool in patients with Parkinson’s disease.^14^
  ■ It has a sensitivity of between 79% and 100% and the specificity is between 67% and 80%.^13^
- Center for Epidemiologic Studies Depression Scale (CES-D)
  ■ This tool takes less than 5 min to administer.
  ■ It can be used in patients with neurocognitive disorders who have a Mini-Mental Status Examination score > 19.
  ■ It has a sensitivity of 85% and a specificity of 64%.^15^

### Differential diagnoses

Although LLD is a common disorder in elderly people, it is important to be aware of potential differentials based on clinical suspicion (Box 1).

BOX 1 Possible differential diagnoses for late-life depression

**Table:** 

**Medical conditions:**
Central nervous system (CNS) – neurocognitive disorders secondary to medical comorbidities, that is, vascular, Parkinson’s disease, neoplastic lesions.
Endocrine disorders – hyperthyroidism, hypothyroidism, hyperparathyroidism.
Infectious and inflammatory disease – HIV-related disorders, autoimmune disorders like Systemic lupus erythematosus (SLE) or scleroderma.
Paraneoplastic syndromes
Sleep disorders – obstructive sleep apnoea.
**Other related psychiatric diagnoses:**
Bipolar disorder, anxiety disorder, post-traumatic stress disorder, adjustment disorder, acute stress disorder.
**Psychosocial adversity:**
Grief, bereavement, and financial loss or retirement
**Substance use, dependence or withdrawal**
**Iatrogenic causes:**
Beta-blockers
H2 receptor blockers
Sedatives
Certain chemotherapy agents
Steroids
Centrally acting antihypertensives

## Management

An understanding of underlying risk factors and pre-emptive screening may assist in the possible prevention and early detection and management of LLD. An integrated collaborative treatment model is preferred as it is associated with the best treatment outcomes. Once a multidimensional diagnosis is made, an interdisciplinary structured management plan must be put in place, which includes a biopsychosocial approach, ongoing symptom monitoring and patient follow-up.

## Pharmacological management

### Key concepts when prescribing in the elderly

When prescribing psychotropics in the elderly, the family physician should have an understanding of the pharmacokinetic and pharmacodynamic changes in this age group. Elderly people are also more likely to have a concurrent chronic medical illness and are possibly on other medical treatments. Potential drug–drug interactions and adverse effects must remain at the forefront of the clinician’s mind when selecting appropriate pharmacotherapy. With this in mind, when prescribing in the elderly, start at the lowest possible dose and up titrate medication slowly, monitoring regularly for side effects.

### Specific psychotropic agents

**FIGURE 2:**
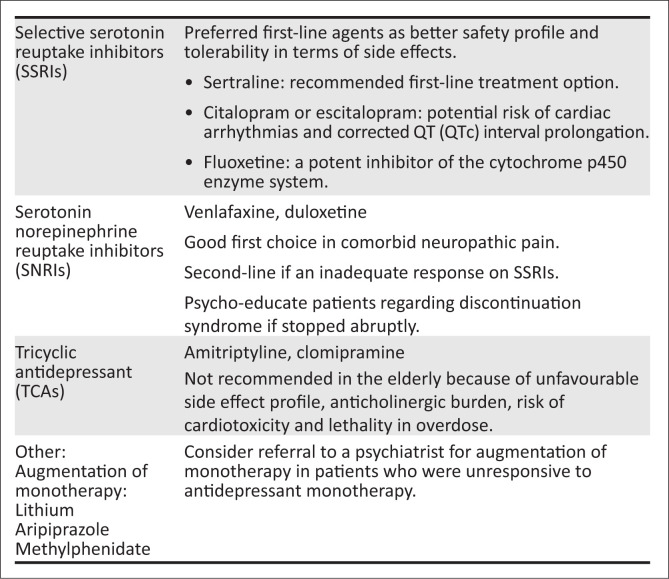
Prescribing of specific psychotropic agents.

Figure 2 summarises preferred evidence-based prescribing of psychotropics in the elderly.

### Nutritional modification

There is a paucity of robust good-quality longitudinal studies on the impact of nutritional deficiencies on mental health, although it would be reasonable to assume that just as nutrition has an impact on physical illness, so too should it have an impact on mental illness. Evidence supports that folate and omega−3 fatty acids may be involved in aetiological factors leading to LLD. There is some evidence to support vitamin D supplementation in patients with deficiency to alleviate symptoms of depression.^16^ It is important to note that nutritional modification and/or supplementation is often used as adjunctive therapy and is not suitable as monotherapy for moderate to severe depression. It is important to familiarise oneself with emerging CAMS in the treatment of depression as this evidence base grows.

### Risk factor control

Addressing modifiable risk factors may assist in alleviating depressive symptoms and preventing symptom progression and sequelae. The family physician should prioritise the management of vascular disease, the control of comorbid illnesses and the prevention of obesity.

## Nonpharmacological management

Interventions considered as nonpharmacological management of LLD may be preferable in mild LLD or used as adjuncts in more severe cases. These include psycho-education, physical activity and behavioural activation and psychotherapy.

### Psycho-education

Psycho-education includes explaining why the diagnosis is being made with objective evidence, educating on signs of relapse and important risk-assessment red flags. Therapeutic approaches should be explored and patient preferences considered. The support of caregivers is paramount in monitoring symptoms and facilitating adherence, as well as identifying caregiver burnout and identifying the need for alternative sources of support.

### Physical activity

Structured physical exercise results in the release of endorphins and may improve cardiovascular health, cognition, feelings of well-being and social engagement.^17^ Meta-analysis recommends the use of structured, supervised exercise programs 3–4 times per week for 45–60 min over a 10–14 week period, with monitoring of response. Intensity needs to be dictated by the individual’s motivation and physical capacity, and even low-intensity exercise may offer benefits for mild to moderate depression.^17^

### Psychotherapy

Psychotherapy should be explored in those patients with mild or moderate LLD, if it is the patient’s preference, in cases with significant medical conditions or potential drug–drug interactions or as an adjunct to pharmacotherapy.

Meta-analysis shows that psychotherapy and pharmacotherapy have comparable efficacy in LLD.^18^ This must be balanced with an understanding of South Africa’s context and the accessibility to psychotherapy in our resource-limited environment. Effective psychotherapies include problem-solving therapy, cognitive behavioural therapy, interpersonal therapy and acceptance and commitment therapy.^19^

## When to refer

Mild to moderate LLD can be managed appropriately by the family physician. Indications for referral to a psychiatrist include those patients where there is a concern regarding:

- having had a poor response (persistent depressive symptoms) on treatment
- need for augmentation
- unmasking of hypomania or mania
- psychotic symptoms
- concerns regarding suicide risk
- intolerable side effects to medication
- multiple comorbidities
- neurocognitive disorder, including mild cognitive impairment.

## Conclusion

Late-life depression is an important disorder for the family physician to be aware of and feel a sense of mastery in diagnosing and managing. It is often underdiagnosed due to its atypical presentation, overlap with neurocognitive disorders and being masked by somatic presentations. An understanding of risk factors, insights as to how to make the diagnosis and appropriate management options allows for the effective treatment of this prevalent disorder.
